# Social and Poor-Law Problems

**Published:** 1907-07-27

**Authors:** 


### SOCIAL AND POOR-LAW PROBLEMS.

### PAUPERS WITH PROPERTY.

A pauper is supposed to be a destitute person, but Poor-law officials know that very often an inmate of the workhouse has a private hoard. By right the inmate should, in such a case, pay to the extent of his liability for the relief he is receiving. But it is not so easy as might be imagined to manage this. When a pauper makes up his mind to go into the workhouse he invariably gives all he has to his friends or relatives. That is understood, but we find pensioners and other persons who are in receipt of a regular income—though perhaps one insufficient for their entire maintenance—living in the workhouse and paying nothing. They go out when the pension or dividend comes due, spend it, or bank it, or give it away, and in a few days come back destitute as before. Thus, in St. George's (Hanover Square) Union a pauper boasted in a rash moment that he had an income of 14s. a week from money invested in Consols. This man has been an inmate of the workhouse for 20 years, and has had this income all the time. But he goes out for a little, drinks his income, and then comes back destitute to the workhouse, where he cannot legally be refused admittance. Another pauper in the same workhouse, a woman, was found to have more than £100 hidden in her clothes. This hoard, being discovered, can be used for the pauper's maintenance, but as yet no plan has been devised that can touch the other case.

Recently, a man who had been an inmate of the Birkenhead Union for five years, came into some money, and the guardians of the Union claimed from him a sum equivalent to the cost of his maintenance during that time. The man contested the claim, on the ground that there had been no contract between him and the guardians. He argued that the guardians maintained him as a statutory duty, and that he had never undertaken to repay them. Happily, the court decided that, while the guardians were bound to maintain a person who was destitute, the pauper was bound to repay the cost if ever he had the means. One can fully sympathise with the man feeling aggrieved that a great part of his legacy was to be taken from him, but it would open the door to endless fraud if a pauper who inherited money were allowed to walk off without a thought of repayment. With regard to pensions and the like, the law should be placed on such a footing that the guardians should be able to put in a claim for arrears of maintenance, and have the money paid over to them.

